# A Comparative Analysis of Aerosol Optical Coefficients and Their Associated Errors Retrieved from Pure-Rotational and Vibro-Rotational Raman Lidar Signals

**DOI:** 10.3390/s21041277

**Published:** 2021-02-11

**Authors:** José Alex Zenteno-Hernández, Adolfo Comerón, Alejandro Rodríguez-Gómez, Constantino Muñoz-Porcar, Giuseppe D’Amico, Michaël Sicard

**Affiliations:** 1 CommSensLab, Deptment of Signal Theory and Communications, Universitat Politècnica de Catalunya (UPC), 08034 Barcelona, Spain; comeron@tsc.upc.edu (A.C.); alejandro@tsc.upc.edu (A.R.-G.); constan@tsc.upc.edu (C.M.-P.); msicard@tsc.upc.edu (M.S.); 2 Instituto Nacional de Astrofísica, Óptica y Electrónica (INAOE), 72840 Puebla, Mexico; 3 Consiglio Nazionale delle Ricerche, Istituto di Metodologie per l’Analisi Ambientale (CNR-IMAA), Tito Scalo, 85050 Potenza, Italy; giuseppe.damico@imaa.cnr.it; 4 Ciències i Tecnologies de l’Espai-Centre de Recerca de l’Aeronàutica i de l’Espai, Institut d’Estudis Espacials de Catalunya (CTE-CRAE/IEEC), Universitat Politècnica de Catalunya (UPC), 08034 Barcelona, Spain

**Keywords:** Raman lidar, pure rotational lines, vibro-rotational lines, theory, experiment, daytime, extinction retrieval

## Abstract

This paper aims to quantify the improvement obtained with a purely rotational Raman (PRR) channel over a vibro-rotational Raman (VRR) channel, used in an aerosol lidar with elastic and Raman channels, in terms of signal-to-noise ratio (SNR), effective vertical resolution, and absolute and relative uncertainties associated to the retrieved aerosol optical (extinction and backscatter) coefficients. Measurements were made with the European Aerosol Research Lidar Network/Universitat Politècnica de Catalunya (EARLINET/UPC) multi-wavelength lidar system enabling a PRR channel at 353.9 nm, together with an already existing VRR (386.7 nm) and an elastic (354.7 nm) channels. Inversions were performed with the EARLINET Single Calculus Chain (SCC). When using PRR instead of VRR, the measurements show a gain in SNR of a factor 2.8 and about 7.6 for 3-h nighttime and daytime measurements, respectively. For 3-h nighttime (daytime) measurements the effective vertical resolution is reduced by 17% (20%), the absolute uncertainty (associated to the extinction) is divided by 2 (10) and the relative uncertainty is divided by 3 (7). During daytime, VRR extinction coefficient is retrieved in a limited height range (<2.2 km) preventing the SCC from finding a suitable calibration range in the search height range. So the advantage of using PRR instead of VRR is particularly evidenced in daytime conditions. For nighttime measurements, decreasing the time resolution from 3 to 1 h has nearly no effect on the relative performances of PRR vs. VRR.

## 1. Introduction

Aerosol remote sensing with elastic-only lidar instruments has the drawback that the effects of the aerosol backscatter coefficient and the aerosol extinction coefficient appear in an indistinguishable way—without more or less plausible further assumptions—in the received signal [1]. To get over this issue and to be able to determine independently the two optical parameters, several solutions exist, one of the most widely extended being the implementation of a channel measuring the backscattered radiation shifted by Raman effect from an abundant atmospheric species: diatomic Nitrogen (N_2_) and diatomic Oxygen (O_2_), with well-defined proportion in the atmospheric composition [2,3]. The principle lies in that, for a purely molecular atmosphere, the law followed by the molecule-specific Raman-shifted radiation collected by the lidar receiver is known, as it only depends (assuming that it does not fall in the absorbing spectrum of an atmospheric gas) on the species number concentration and the molecular scattering; hence, departures from this known law can be related to the extinction introduced by the aerosols.

This technique has been used for nearly three decades [4,5,6]. The most common implementation is the combination of elastic signals and their associated N_2_ Vibro-Rotational Raman (VRR) signal, for example, 355 nm (elastic) and 387 nm (Raman), or 532 nm (elastic) and 607 nm (Raman). Because the realtively high difference between the excitation wavelength and the Raman-shifted one, the use of the VRR spectrum makes it easy to provide the needed rejection to the excitation wavelenght in the Raman channel. However, in most cases this technique is limited to nighttime measurements because the noise induced by the daytime background solar radiation passing through the Raman-channel interference filter swamps the Raman signal provided by the low differential backscatter cross-section of the VRR spectrum [7]. Moreover, the significant wavelength shift of the VRR spectrum with respect to the excitation wavelength introduces an additional source of uncertainty, as an assumption about the spectral dependence of aerosol extinction is needed for the retrieval of both the extinction and backscatter coefficients [3].

To overcome these limitations pure rotational Raman (PRR) scattering can be used instead of vibro-rotational scattering. While PRR scattering has been widely used in laser remote sensing for the measurement of atmospheric temperature [8], its use for aerosol remote sensing is more recent and thus less common [7,9,10,11] and has been prompted by the availability of compact interference filters able to select a suitable group of N_2_ and O_2_ PRR spectrum lines, while providing enough rejection at the close excitation wavelength. The small spectral separation between the elastic and PRR lines implies that the spectral dependence of aerosol extinction has virtually no effect on the retrieval of the extinction and backscatter coefficients. Also, the total differential backscatter cross-section of both N_2_ and O_2_ PRR is greater than that of vibro-rotational scattering. N_2_ PRR measurements have been used successfully to retrieve extinction coefficients at 532 [7] and 1064 nm [11], and the first tests have been performed at 355 nm [11].

In this paper, we present simultaneous PRR and VRR measurements at 355 nm in order to evaluate the daytime/nighttime performances of both channels in different conditions of aerosol load. The European Aerosol Research Lidar Network/Universitat Politècnica de Catalunya (EARLINET/UPC) multi-wavelength lidar system [12,13] was modified for this purpose. An energy budget is presented in relative terms between both PRR and VRR channels and quantifies the gain in signal-to-noise ratio for day and nighttime operations. Then the aerosol optical properties are retrieved from both PRR and VRR configurations with the Single Calculus Chain (SCC) of the European Research Infrastructure for the observation of Aerosol, Clouds, and Trace Gases (EARLINET/ACTRIS). Differences between both techniques are analyzed in terms of vertical resolution, error bars and day/night conditions.

## 2. Materials and Methods

### 2.1. Pure Rotational and Vibro-Rotational Raman Spectra Calculation for N_2_ and O_2_

The result of energy interaction between an electromagnetic field and an atomic system is characterized by elastic and inelastic (Raman being one of them) processes. In the case of the Raman process, the scattered radiation is accompanied of an energy loss (Stokes wing of the Raman spectrum) or gain (anti-Stokes wing of the Raman spectrum). These changes, which can be observed as a frequency shift of the scattered photons, are directly related to the vibrational and rotational quantized energy states of the atomic system or molecule [14]. The vibrational-rotational Raman spectrum is defined by a distribution of spectral lines centered on a Q branch that possesses the highest intensity and two side branches: O and S, occurring at lower (Anti-stokes) and higher (Stokes) wavelengths, respectively. The final distribution of the vibrational-rotational Raman lines depends strictly on temperature T and excitation wave number ν_0_ = 1/λ_0_ [15].

A complete analysis of the Differential Backscatter Cross-Sections (DBCS) calculation for both PRR & VRR contributions from N_2_ and O_2_ is shown in Appendix A of this work.

The total contributions of the Raman backscatter spectra are estimated by the summation of the lines of the molecule’s spectral branches: O, Q & S (VRR) and O & S (PRR) weighted by their respective atmospheric concentration: 0.7808 for N_2_ and 0.2095 for O_2_ [14,16,17].

**Figure 1:**
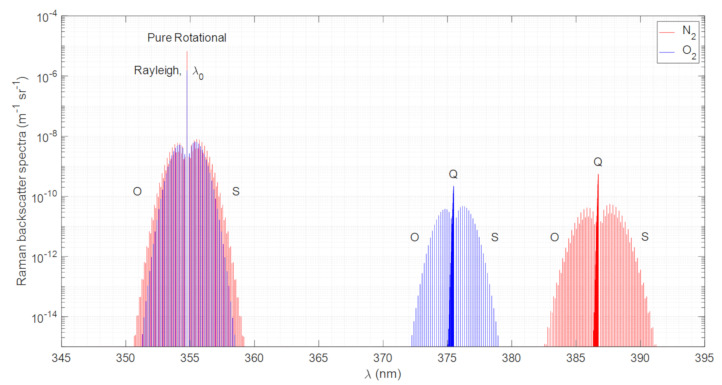
Pure rotational and vibro-rotational Raman backscatter spectra intensities calculated for diatomic Nitrogen (N_2_) and diatomic Oxygen (O_2_). Excitation wavelength: 354.76 nm. The spectra shown correspond to a 300 K temperature and to a 1013 hPa pressure.

The lidar system used in this work employs a Nd:YAG (Neodymium-doped Yttrium Aluminum Garnet; Nd:Y_3_A_l5_O_12_) laser with a fundamental wavelength at 1064.28 nm. The second and third harmonics are thus expected at 532.14 nm and 354.76 nm, respectively [18]. Considering the third-harmonic excitation wavelength, λ_0_, of 354.76 nm and a temperature of 300 K, the computed Raman backscatter spectra for the PRR and the VRR contributions of N_2_ and O_2_, according to the Appendix A formulas, is shown in Figure 1 for 300 K temperature and standard sea-level pressure. The total contribution of the resulting intensities, calculated by the summation of the spectral lines for the PRR (O & S branches) and the VRR (O, Q & S branches) spectra, are given in Table 1. The ratio between PRR and VRR intensities from the backscatter spectra, PRR/VRR is 21.21 and 37.03 for N_2_ and O_2_, respectively.

In practice one cannot take advantage of the whole PRR spectrum because it encompasses the elastics return. Moreover, the selection of lines to pass through the interference filter must be such that sum of their DBCS is, to the maximum possible extent, insensitive to the temperature. Nevertheless, the overall cross section of that selection of N_2_ and O_2_ PRR spectrum lines still outweighs that of the total VRR spectrum.

### 2.2. Optical Design and PRR Spectral Filtering

**Figure 2:**
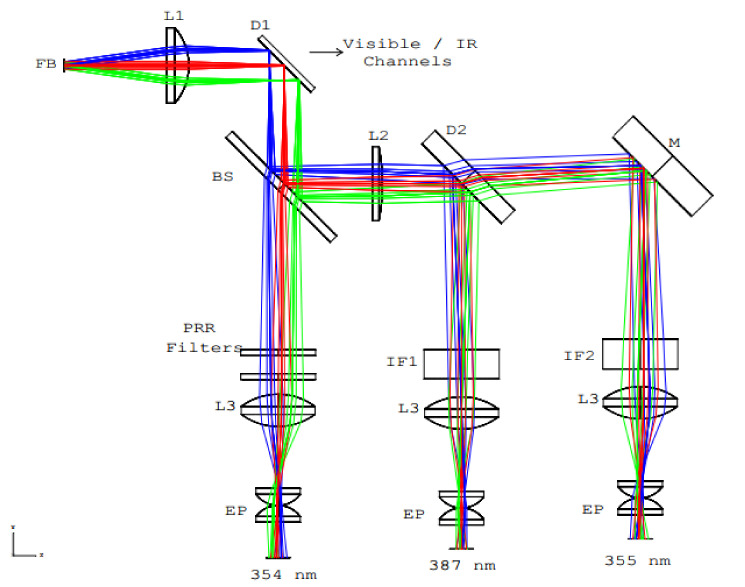
Zemax optical layout. FB stands for fiber bundle, L for lens, D for dichroic mirror, BS for beam splitter, EP for eyepiece, IF for interferential filter and M for mirror. Blue/red/green rays have a divergence of 15°, 0° and −15°. 15° corresponds to the numerical aperture of the fiber bundle.

The EARLINET/UPC multi-wavelength lidar system was designed with a six channel wavelength-selection subsystem for the detection of Ultraviolet (UV), visible and Infrared (IR) backscatter returns [12,13]. The light collected by the telescope is transported by an optical fiber bundle (designed as FB in Figure 2) to the wavelength-selection subsystem. The optical setup of the UV branch has been especially redesigned for this study as shown in Figure 2. The main characteristics of the used optical elements are given in Table 2. Note that no attempt has been made to optimize the power reaching the Raman channels (e.g., by using a dichroic beam splitter, instead of the plain beam splitter labelled (BS) in Figure 2), because we were relying on a previous existing setup [12].

Using a first dichroic beam splitter (D1) UV and Visible-IR branches of the collected backscattered radiation are divided. After the first reflection on D1, the UV portion of the spectrum is divided into three channels. The first 50/50 beam splitter (BS) divides the incoming light into equal portions. Lens L2 corrects the divergence of the rays for the channels at 354.76 and 386.7 nm (N_2_ VRR spectra center wavelength). D2 reflects high UV wavelengths of the incoming radiation, redirecting this portion of the spectra to the 386.7 nm channel, and transmits the low UV wavelengths, which is reflected by a mirror (M) to the 355-nm channel. Lenses L3 and eye-pieces (EP) are used to, respectively, collimate and equally spread the radiation on the surface of the photomultiplier tube (PMT) detectors.

**Figure 3:**
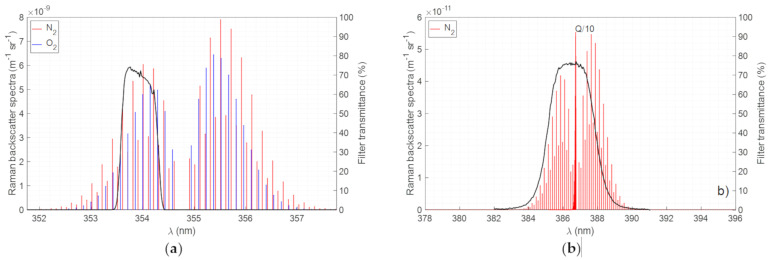
(**a**) N_2_ and O_2_ pure rotational Raman spectra (left axis) and filter transmittance (right axis) (**b**) N_2_ vibro-rotational Raman spectrum (left axis) and filter transmittance (right axis). The spectra shown correspond to a 300 K temperature and to a 1013 hPa pressure.

Elastic and VRR signals are optically filtered by, respectively, 1-nm and 3-nm bandwidth interference filters (see Table 2). As N_2_ atmospheric concentration is larger than O_2_, its total differential cross section is also larger (see Table 1). For this reason, the vibro-rotational channel is set to that of nitrogen. For the pure rotational contribution, two interference filters set in cascade are also used to minimize the crosstalk from the elastic channel. Between both O and S branches, only the anti-Stokes branch (O-branch) is considered to avoid aerosol fluorescence effects. Also, due to the technological impediments that hinder the effective elastic return rejection. For the fabrication of the PRR filters, a special request was sent to the manufacturer (Alluxa, North Laughlin Rd., Santa Rosa, CA 95403, USA) for two extremely narrow and steep interference filters with Center Wavelength (CWL) at 353.9 nm, bandwidth <1 nm, transmission at peak >60% and Optical Density (OD) of 4 (OD4) at 354.7 nm. The company delivered two filters with CWL at 353.9 nm, bandwidth <0.8 nm, transmission at peak >80% and OD4 at 354.7 nm. An effective suppression of the elastic wavelength is achieved by cascading these two interference filters although no attempt has been made to compare the response of the two cascaded filters to that of a single one. The resulting equivalent transmittance, assuming the response of the two-filter cascade is the product of the individual responses, is shown in Figure 3a, while the transmittance of the VRR filter is shown in Figure 3b. The corresponding differential backscatter cross-section are also overlapped on these figures.

### 2.3. PRR vs. VRR Channel Numerical Comparison

This sub-section is devoted to the estimation of the overall gain ratio between PRR and VRR channels i.e., Γ = PRR/VRR, particularized for the used system. Γ, can be defined as:

(1) $$\Gamma =\left(\frac{ECDS_{PRR}}{EDCS_{VRR}}\right)\left(\frac{OPL_{PRR}}{OPL_{VRR}}\right)\Gamma_{PMT}$$

where EDCS is the effective differential cross section, OPL is the optical path loss (excluding the interference filters) and Γ*_PMT_* is the gain ratio of the PMTs.

The EDCS is calculated by the summation of all the molecular differential cross-section lines *σ^i^_PRR,VRR_*(*J*,*T*) (see Appendix A) of N_2_ and O_2_, in the case of the PRR spectrum, and N_2_ for the VRR spectrum, weighted by the respective atmospheric concentration *N_i_* (0.7808 for N_2_ and 0.2095 for O_2_) and the filter transmittance *ξ*(*λ_i,j_*):

(2) $$EDCS_{PRR,VRR}\left(T\right)=\sum_{i}\sum_{j}N_{i}\sigma_{PRR,VRR}^{i}\left(J,T\right)\xi \left(\lambda_{i,j}\right)$$

Subindexes *i* = 1, 2 correspond to the molecules N_2_ or O_2_, *J* stands for the rotational quantum number.

The estimated EDCS for the PRR and the VRR channels are given in Table 3. The resulting *EDCS_PRR_*/*EDCS_VRR_* ratio is 8.2.

The optical transmittance budget for PRR and VRR channels is also estimated in Table 3. All the optical elements situated on the optical path from the output of the fiber bundle to the PMT detectors are considered, except for the interference filters, which are already considered in the calculation of EDCS. The total OPL is 36% and 32% for PRR and VRR, respectively. These two values are very similar and indicate that both channels suffer similar optical losses along their respective optical path. With these numbers one can calculate the ratio (*EDCS_PRR_*/*EDCS_VRR_*) (*OPL_PRR_*/*OPL_VRR_*) which is equal to 9.2.

**Figure 4:**
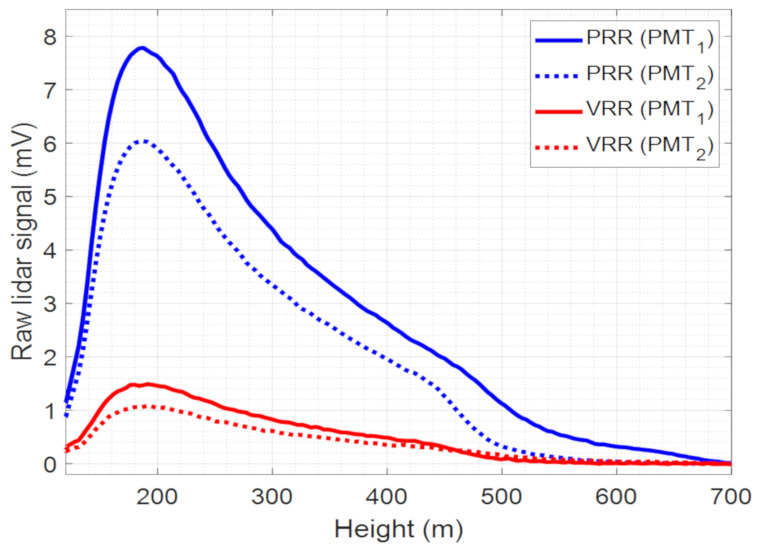
Raw lidar signal at the Photomultiplier tube (PMT) outputs over a 50 Ω load of PRR and VRR channels in nominal condition (PRR → PMT1; VRR → PMT2) and permuted (PRR → PMT2; VRR → PMT1). The background noise offset has been subtracted to the lidar signal.

Finally, to consider a possible gain difference between the detectors, measurements with each PMT in its nominal position and with the PMTs swapped were made. In nominal conditions, PMT1 and PMT2 are the PMTs of the PRR and VRR channels, respectively. In the first measurement (nominal), PMT1 detected the signal of the PRR channel and PMT2 of the VRR one. In the second measurement (permuted), PMT2 detected the signal of the PRR channel and PMT1 of the VRR one. The background-subtracted lidar signals are shown in Figure 4. For the same channel, PMT1 gives a higher signal than PMT2 and thus has a higher gain. The PMT gain ratio, Γ_PMT_, was calculated by dividing the signals PMT1 over PMT2 averaged in the range [120, 700 m] for both PRR and VRR channels and taking the mean value of both ratios. We find Γ*_PMT_* = 1.32.

We can now calculate the overall gain ratio between PRR and VRR channels due to differences in interference filter transmittances, optical path losses and PMT gains [Equation (1)] being Γ = 12.14. This ratio not only estimates the total contribution of the EDCS of the PRR and VRR signals, but also contains experimental setup parameters (optics and detector gains), it is considered in the following a reference gain ratio to which real atmospheric signals can be compared to.

### 2.4. Temperature Analysis of the Effective Differential Cross-Section (PRR)

Pure rotational Raman lidars are commonly used for atmospheric temperature sensing since lines in the rotational spectra are sensitive to temperature [8]. This sub-section is devoted to the estimation of the temperature dependence of the PRR EDCS to verify that it can be neglected.

We computed *EDCS_PRR_*(*T*) [Equation (2)] variation in a conservative range of temperatures from 200 to 300 K. According to the US Standard atmosphere [19], 300 K corresponds to ground level and 200 K is reached at altitudes higher than 10 km. Considering T_0_ = 300 K as a reference, the relative variation of *EDCS_PRR_*(*T*) due to temperature has been estimated by analyzing the ratio:

(3) $$\psi \left(T\right)=\frac{EDCS_{PRR}\left(T\right)-EDCS_{PRR}\left(T_{0}\right)}{EDCS_{PRR}\left(T\right)}$$

**Figure 5:**
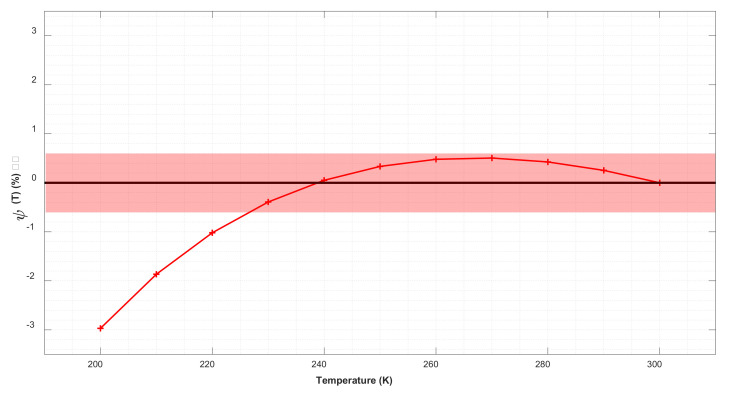
Relative temperature variation of *ψ*(*T*). Reference temperature T_0_ = 300 K. The shaded area indicates relative errors in the range [−0.5, 0.5].

The results, shown in Figure 5, demonstrate that the relative variation of *EDCS_PRR_*(*T*) due to temperature does not exceed 3% in the 300 K–200 K temperature range and are even smaller than 0.5% for 230 < T < 300 K, i.e., below 10 km altitude.

To quantify the temperature-induced variations for the estimation of backscatter and extinction coefficients, we rely on the theory developed in [20] and afterwards employed for pure rotational Raman lidar applications by [7,11]. In these works, expressions for the calculation of backscatter and extinction coefficients are developed considering the temperature dependency:

(4) $$\beta_{aer}\left(z\right)=-\beta_{mol}+\beta_{mol}\left(z_{0}\right)\cdot \frac{P_{\lambda_{R}}\left(z_{0}\right)P_{\lambda_{0}}\left(z\right)N\left(z\right)}{P_{\lambda_{0}}\left(z_{0}\right)P_{\lambda_{R}}\left(z\right)N\left(z_{0}\right)}\cdot \frac{\sigma^{eff}\left(z\right)}{\sigma^{eff}\left(z_{0}\right)}$$

(5) $$\alpha_{aer}\left(z\right)=\frac{1}{2}\frac{d}{dz}\ln\left[\frac{N\left(z\right)}{P_{\lambda_{R}}\left(z\right)z^{2}}\right]+\frac{1}{2}\frac{d}{dz}\ln\left[\sigma^{eff}\left(z\right)\right]-\alpha_{mol}\left(z\right)$$

Subscripts aer and mol stand for aerosol and molecular contributions, *λ*_0_ and *λ_R_* are excitation and Raman wavelengths respectively, and N is the molecular density. *σ^eff^* (*z*) is the temperature-varying *EDCS_PRR_* as a function of height.

**Figure 6:**
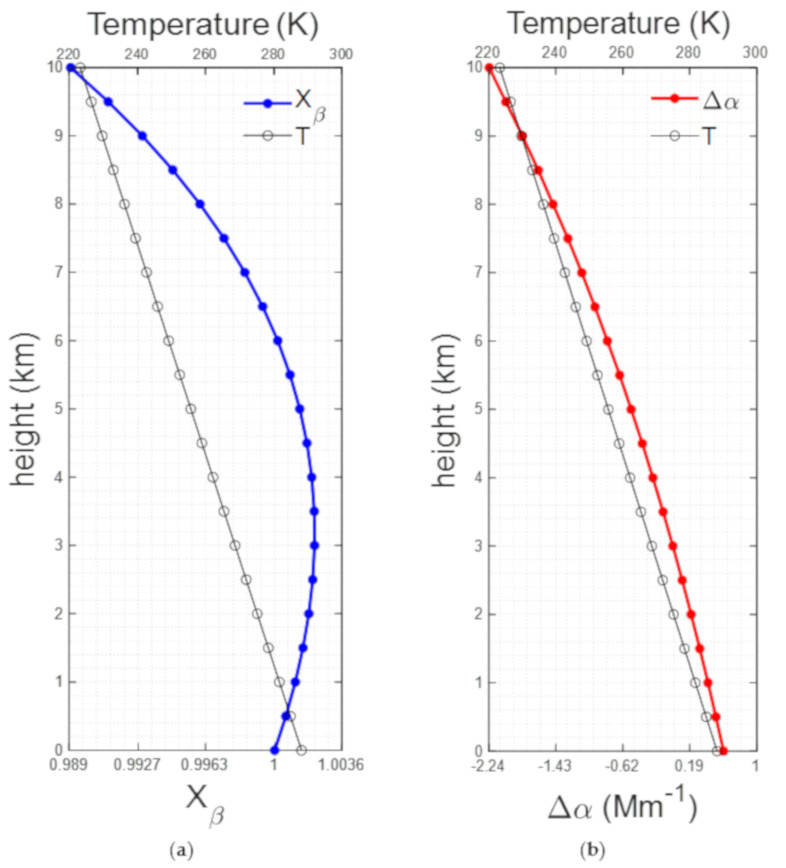
(**a**) Temperature-induced variation for the backscatter coefficient (**b**) temperature-induced error for the extinction coefficient. Temperature profile from U.S. Standard Atmosphere.

Temperature-induced variation for the backscatter coefficient can be estimated by computing the temperature height variation of *σ^eff^* (*z*)/*σ^eff^* (*z*_0_) (henceforth identified as *X_β_*) in Equation (4), reference height *z_0_* was chosen to be 0 km. Temperature-induced error for the extinction coefficient was computed by the temperature height variation of the term (½)(*^d^*/*_dz_*)ln[*σ^eff^* (*z*)] (henceforth identified as Δα) in Equation (5). For both calculations the temperature range was 288.15 to 223.25 K corresponding to a height range of 0 to 10 km according to the U.S. Standard Atmosphere model [19]. Results are shown in Figure 6. These computations show a temperature-induced variation for *X_β_* that does not exceed 1%. For the extinction, the absolute error variation is less than 1 Mm^−1^.

The authors in [7] performed a similar analysis for *ψ*(*T*) having found less than 1% of temperature dependency for the EDCS (*λ*_0_ = 532 nm). They reported a temperature-induced variation for the backscatter coefficient less than 1% and for the aerosol extinction coefficient error less than 2 Mm^−1^. In reference [11] (*λ*_0_ = 354.75 nm), the authors report a temperature variation for the backscatter coefficient was less than 4%. The extinction coefficient error is reported with an always negative variation of up to −1.6 Mm^−1^. Reference [21], *λ*_0_ = 1064 nm, reports a 4% of temperature dependence of the EDCS. All these results are shown in Table 4. Extinction relative errors were estimated for an aerosol load of 100 Mm^−1^ in all the references. This temperature analysis and comparative allows us to neglect changes related to the temperature influence in the PRR backscattered signal.

## 3. Results

Two nighttime and one daytime measurements are analyzed in this work. The day and time they were performed are indicated in Table 5, as well as some atmospheric parameters taken from Aerosol Robotic Network (AERONET) data, such as the aerosol optical depth at 440 nm, AOD_440_, and the Ångström exponent calculated from the wavelength pair (440 nm, 675 nm), AE_440–675_. The first nighttime measurement (N1) lasted for 3 h. The second nighttime study case (N2) corresponds to the first hour from N1. This 1-h-averaged measurement was chosen to examine the effect of temporal resolution on the quality of the retrievals. The daytime measurement (D1) lasted for 3 h as well. In N1 and N2 aerosols were of local origin and accumulated mostly in the planetary boundary layer (PBL): AOD_440_ is equal to 0.15 and AE_440–675_ to 1.27. In D1 most of the locally originated aerosols are in the PBL; mineral dust particles are present in the free troposphere up to 4.5–5 km. This situation is associated with a higher AOD_440_ (0.26) and a low AE_440–675_ (0.71).

### 3.1. PRR vs. VRR: Signal and Signal-to-Noise Ratio Comparisons

To get a quantitative comparison between PRR and VRR preprocessed (glued) signals, Signal-to-noise ratio (SNR) was calculated computing estimators of the mean value μ and the standard deviation *σ* over a 9-samples continuous sliding interval of the signal. SNR is estimated as *μ*/*σ*. To smooth the SNR calculation, a model was fitted to the *σ* estimator [22,23]. In photon-induced current devices, e.g., photomultiplier tubes, SNR can be calculated as [24]:

(6) $$SNR=\frac{P_{s}}{\sqrt{P_{q}P_{s}+P_{q}P_{b}+NEP^{2}B}}$$

where *P_s_* is the signal power, *P_b_* is the background radiation, *P_q_* is the quantum noise power defined as: *P_q_* = 2*FhcB*/*ηλ*, *B* is the photoreceiver electrical bandwidth, and *NEP* is the noise equivalent power of the receiver. Values of the mean and the standard deviation estimators are related as *μ = P_s_* and *σ* = (*P_q_P_s_* + *P_q_P_b_* + *NEP*^2^*B*)^1/2^, respectively.

The standard deviation *σ*, can be expressed as: *σ* = *(K*_1_*μ* + *K*_2_)^1/2^ with *K*_1_ = *P_q_* and *K*_2_ = *P_q_P_b_* + *NEP*^2^*B*. To suppress the contribution of high varying near ranges, which could “fool” the overall fit, a logarithmic fitting is considered: log(*σ*) *=* ½log(*K*_1_*μ* + *K*_2_). The fitting was based on a nonlinear least-squares method [25,26] that retrieves the *K_i_* coefficients that best fit the function ½log(*K*_1_*μ* + *K*_2_) to the estimator *σ* starting from first guess values of *K*_1_ and *K*_2_ and the previous estimated values of *μ* and log(*σ*). SNR was then re-calculated with the fitted *σ*.

**Figure 7:**
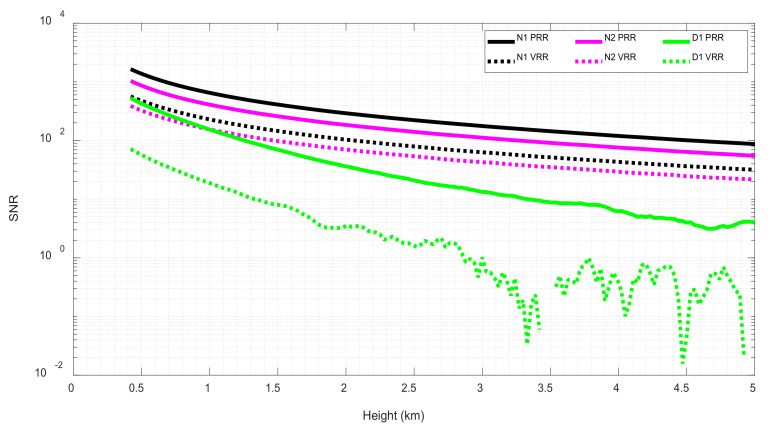
PRR and VRR Signal-to-noise ratio.

Figure 7 shows the SNR estimated for the glued signals in a 0.5 to 5 km range. First 500 m were omitted to avoid overlap effects. For all three study cases, SNR for the PRR contribution (henceforth called SNR_PRR_) plotted in continuous lines, are higher than the respective SNR from the VRR (SNR_VRR_), plotted in dotted lines. For nighttime cases the enhancement factor, which essentially is the ratio between SNR_PRR_ and SNR_VRR_, is quite constant for all the range, being 2.8 for N1 case and 2.6 for N2. Daytime case SNR_PRR_ is quite constant for most of the interval. SNR_VRR_ calculation kept variations despite the fitting process for the estimation of *σ*. Nevertheless, the enhancement factor of SNR_PRR_ over SNR_VRR_ signal is about 7.6 for most of the range. It is important to note that background radiation for the VRR channel (approximately 3-nm wide filter) is about three times larger than for the PRR channel (approximately 1-nm wide filter), which offers an added advantage in terms of SNR in daytime operation.

To verify the reliability of the calculation for these enhancement factors, estimated with the fitted model for *σ*, this section focusses on the comparison between SNR estimation against real signals gain. For nighttime conditions, background radiation *P_b_*, as well as the noise equivalent power *NEP*, can be neglected from Equation (6). Therefore, SNR estimation may be expressed as:

(7) $$SNR=\frac{\mu }{\sqrt{P_{q}\mu }}=\frac{\sqrt{\mu }}{\sqrt{P_{q}}}$$

The enhancement factor may be calculated as: SNR_PRR_/SNR_VRR_ = {(*μ*_PRR_/*μ*_VRR_) × (*P_q_*_VRR_/*P_q_*_PRR_)}^1/2^. Considering that detectors from both channels are almost the same, the second term may be neglected. Therefore, the enhancement factor is proportional to the square root of the averaged signals ratio as SNR_PRR_/SNR_VRR_ = (*μ*_PRR_/*μ*_VRR_)^1/2^. In a 1 to 3 km interval the computed square root of the averaged signal ratios is 2.54 and 2.53 for both N1 and N2 cases. These values work in accordance with the previously estimated enhancement factors of 2.8 and 2.6, differing less than 10% and 5% respectively.

For the daytime case, background radiation contribution predominates over NEP and P_q_ terms, resulting in a SNR estimation expressed as:

(8) $$SNR=\frac{\mu }{\sqrt{P_{q}P_{b}}}$$

The enhancement factor SNR_PRR_/SNR_VRR_, is recalculated as: SNR_PRR_/SNR_VRR_ = (*μ*_PRR_/*μ*_VRR_)∙{(*P_q_*_VRR_/*P_q_*_PRR_)∙(*P_b_*_VRR_/*P_b_*_PRR_)}^1/2^ which essentially is proportional to the averaged signals ratio weighted by the squared root of the quantum noise power and the background radiation ratio for both PRR and VRR contributions. In the 1 to 3 km interval, the computed averaged signals ratio is 8.24. Omitting the fact that the second term is still affecting the relation, the resulting estimation differs less than 10% compared to the fitted enhancement factor (7.6).

Previously, in Section 2.3, an overall reference gain ratio between PRR and VRR signals was estimated as Γ = PRR/VRR = 12.14 [Equation (1)]. To compare the experimental data against this reference value it is necessary to consider the ratio between averaged signals: *μ*_PRR_/*μ*_VRR_, which stands for the realistic gain ratio between PRR and VRR detected signals. For nighttime cases, this ratio is about 6.45 and for daytime case is 8.24. Percentage differences between the experimental and theoretical value of this calculation reaches values of 47% (nighttime) and 32% (daytime). These differences may be explained by the lack of precision in the optical path losses which may be underestimated and also by possible changes of the aerosol load and the background radiation.

### 3.2. PRR vs. VRR: Optical Product Estimation and Comparison of Performances

The retrieval of the aerosol extinction and backscatter coefficients combining PRR and VRR signals with elastic signals has been performed by using the Single Calculus Chain (SCC) from the European Aerosol Research Lidar Network (EARLINET) [27,28,29]. It is the first time that the SCC is used to retrieve aerosol optical products from a combination of PRR and elastic signals. The optical processor module of the SCC is called EARLINET Lidar Data Analyzer (ELDA). ELDA was configured to retrieve backscatter and extinction coefficients at the same effective vertical resolution (product lidar ratio and extinction) using the Raman method [29]. This product also includes the effective vertical resolution, defined at each altitude point, calculated by ELDA to achieve the error goals fixed by the user [29]. As each Raman signal has its own statistical uncertainties the vertical resolution may differ from one measurement to the other. A coarser vertical resolution means that smoothing was more severe, and the retrieved products suffer loss of spatial information. Hence products with finer vertical resolution are more reliable. Only the product lidar ratio and extinction is shown in this work. VRR optical products for the EARLINET/UPC lidar system already exist in the SCC since 2011. VRR retrievals are restricted to only nighttime measurements. Daytime VRR retrievals have been tested in the past but either the SCC failed in performing the inversion or the results were not judged physically meaningful to be used for science purposes. Among many parameters needed in the SCC configuration, five are important for this work. For the VRR channel they are set as:

- wavelengths set to 355 (elastic) and 387 nm (VRR),
- extinction Ångström exponent is set to 1.0,
- low and high range error thresholds are set to, respectively, 10 and 10% (nighttime) and 10 and 50% (daytime),
- detection limits for the backscatter coefficient to 0.1 Mm^−1^ sr^−1^ and extinction to 5 Mm^−1^,
- height range in which ELDA looks for a suitable calibration interval to 4–8 km.

The error threshold and the detection limit, noted respectively Δ_max_ and Δ_DL_ following [29] nomenclature, are thresholds used in the iterative procedure that calculates the vertical smoothing. In an initial step, the optical products are calculated with the maximum allowable vertical smoothing (500 m below 2 km and 2000 m above 2 km); in the following steps the vertical smoothing is reduced until the relative statistical uncertainty becomes larger than the user-defined error threshold or until the absolute uncertainty becomes larger than the user-defined detection limit (see [29] for more details). In daytime, ELDA was not able to successfully retrieve optical products with the VRR signals with a high-range (above 2 km) Δ_max_ of 10%. So, this value was relaxed to 50% for daytime inversions.

For the PRR optical products, the same configuration as that of VRR products was used with the exceptions that wavelengths were set to 355 (elastic) and 354 nm (PRR). Low- and high range error thresholds, as well as detection limits, were kept unchanged. Finally, the statistical uncertainties associated to the optical products are estimated by Monte Carlo or error propagation methodologies [28,29].

To determine the region where most of the aerosols are, we used a simple threshold method on the extinction coefficient retrieved with the PRR signals. The limit to assume a small but non-negligible amount of aerosols was fixed to the detection limit, Δ_DL_, i.e., 5 Mm^−1^. On a monotonically decreasing profile of extinction coefficient, the region where α_aer_ > Δ_DL_ will represent the main surface aerosol layer. In the case of only one aerosol layer, the height at which the extinction coefficient reaches Δ_DL_ should be a relatively good approximation of the PBL height [30].

**Figure 8:**
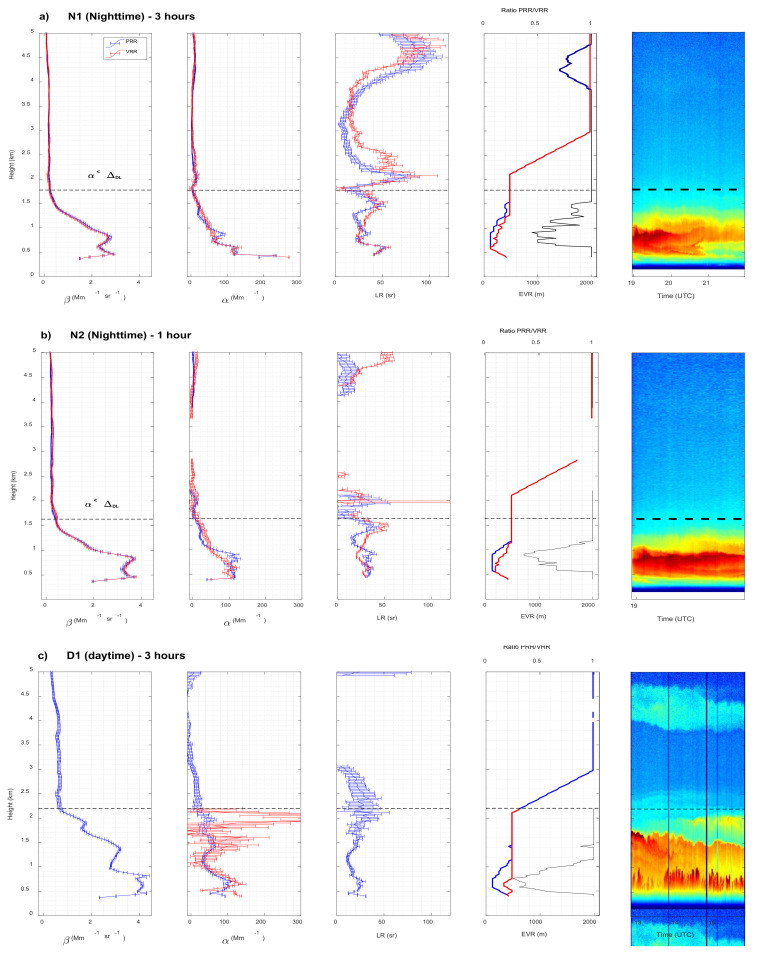
Aerosol backscatter and extinction coefficients, lidar ratio and effective vertical resolution retrieved by the Single Calculus Chain (SCC) from PRR and VRR signals for case (**a**) N1, (**b**) N2 and (**c**) D1. On the plot of the vertical resolution, the ratio of the resolutions PPR to VRR is reported (black line, top axis). Time-height plots of the range-square corrected signal (in arbitrary units) are reported in the far-right plot. In (**a**,**b**) the black horizontal dashed line represents the lowest height for which α < Δ_DL_. In (**c**) it represents the highest height for which $\alpha_{|\mathrm{VRR}}$ is retrieved. The legend in the first plot of Figure 8a applies to all plots.

Figure 8 shows for all three cases the vertical profiles of the aerosol backscatter coefficient, β_aer_, the aerosols extinction coefficient, α_aer_, the aerosol lidar ratio, i.e., the ratio α_aer_/β_aer_, noted LR, and the Effective Vertical Resolution (EVR), for both PRR and VRR retrievals. The time-height plot of the range-square corrected signal (in arbitrary units) is also reported to get an idea of the atmospheric structure. The subscript |PRR or |VRR behind a symbol indicates whether it was retrieved with the PRR or VRR signal, respectively. β_aer_ and α_aer_ with their associated uncertainties, as well as EVR are plotted as is without any post processing. Resulting negative values negative or larger than 100 sr are not represented in Figure 8.

In both nighttime cases, retrieved profiles of β_aer_ and α_aer_ from both pure rotational and vibro-rotational channels are quite similar. While the profiles of N1 are continuous, the retrievals of the 1-h nighttime case (N2) show discontinuities above the main surface aerosol layer related to negative extinction retrievals discarded in the output of the SCC. In case D1 the PRR retrieval is successful and that of the VRR is partly successful: α_aer|VRR_ is retrieved but β_aer|VRR_ is not (Figure 8c). It is worth recalling at this point that VRR retrievals with the EARLINET/UPC lidar system have always been restricted to nighttime conditions because the VRR inversion of daytime optical products either failed or was not judged physically meaningful to be used for science purposes. It is thus a small achievement that a profile of α_|VRR_ was obtained for case D1. By looking at the profile of α_aer|VRR_, the explanation why β_aer|VRR_ is not retrieved is quite straightforward: α_aer|VRR_ is not retrieved above 2.2 km, i.e., EVR is not available in the range 4–8 km in which ELDA looks for a calibration interval for the calculation of β_aer_; in these conditions ELDA is not able to calculate the molecular backscatter coefficient β_mol_(z_0_) at the calibration height upon which depends the retrieval of β_aer_. In case D1, α_aer|PRR_ and α_aer|VRR_ agrees generally well up to 2.2 km. Differences are observed near the surface and in the last 500 m. Near the surface the difference is attributed to different vertical resolution, while between 1.7 and 2.2 km the difference is probably due to the degradation of the SNR in the VRR channel. Above 2.2 km α_aer|VRR_ is not retrieved although aerosols are still present. α_aer|PRR_ is retrieved above 2.2 km, although with increasing error bars and negative (i.e., non-physical) values starting above 3.1 km. The probable dust layer at 4–4.5 km is visible on the profile of β_aer|PRR_ but not on that of α_aer|PRR_. This reveals the limit of the PRR performances in daytime conditions. Since optical products from PRR and VRR retrievals agree well, the quantitative, comparative analysis discussed in the next section is centered on their associated errors.

The profiles of LR can only be compared for both nighttime cases (β_aer|VRR_ is not retrieved in case D1). Above the height at which α_aer_ < Δ_DL_, the comparison is difficult because the low aerosol regime induces low signals and SNR, forcing ELDA to use full vertical resolution (2 km) in most of the upper interval. For that reason, the comparison is made only up to the height at which α_aer_ < Δ_DL_, i.e., 1.78 and 1.63 km for N1 and N2, respectively. Up to those heights, the profiles of LR are very similar between both techniques. A difference can be pointed out in case N1: between 0.8 and 1.5 km LR_|VRR_ variations appear smoother than the ones of LR_|PRR_. The explanation lies in two facts: in this height range, 1) the atmosphere is highly variable (see the time-height plot in Figure 8a) and 2) the vertical resolution of the PRR products is 20 to 40% smaller than that of the VRR products. Both facts yield a smoother profile of α_|VRR_, and thus of LR_|VRR_.

In all cases and at all altitude ranges the ratio EVR_|PRR_/EVR_|VRR_ is smaller than or equal to 1, meaning that the extinction retrieval from PRR signals requires less or equal vertical smoothing than the VRR retrieval. In all cases also, below 2 km, EVR_|VRR_ reaches its maximum value, 500 m, at lower heights than EVR_|PRR_. As seen in the plots of EVR in Figure 8, below 1.5 km, so typically in the PBL height in Barcelona [31], the lowest ratio EVR_|PRR_/EVR_|VRR_ is 44, 36 and 25% for case N1, N2 and D1, respectively. Faced to VRR performances, PRR ones seem enhanced in daytime conditions (lower EVR_|PRR_/EVR_|VRR_ ratio) and seem to decrease with increasing temporal resolution (EVR_|PRR_/EVR_|VRR_ ratio higher for N1 than for N2).

## 4. Discussion

As a quantitative hint of the quality of the measurement the absolute and relative uncertainties were calculated for α_aer_ and β_aer_. As it was mentioned before, ELDA estimates statistical uncertainties via the standard formula of statistical error propagation [28].

**Figure 9:**
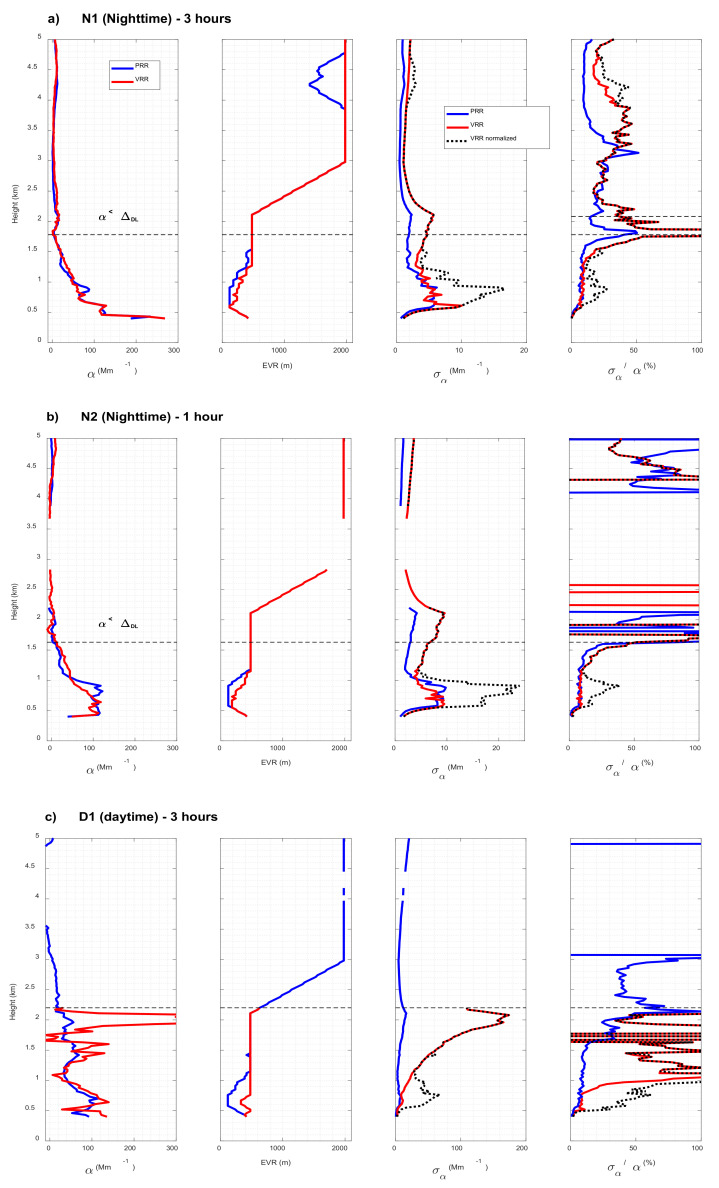
Aerosol extinction coefficient, vertical resolution, absolute and relative errors retrieved by the SCC from PRR and VRR signals for case (**a**) N1, (**b**) N2 and (**c**) D1. In (**a**,**b**) the black horizontal dashed lines represent the lowest height for which α < Δ_DL_. In (**c**) it represents the highest height for which α_|VRR_ is retrieved. “VRR normalized” refers to the magnitude recalculated at or normalized to EVR_|PRR_. The legends in Figure 9a apply to all plots.

**Figure 10:**
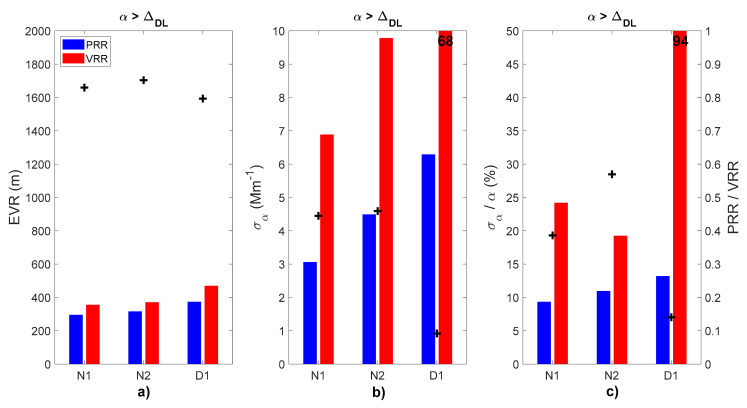
Comparative histograms of layer-mean values of the (**a**) vertical resolution, (**b**) absolute uncertainty and (**c**) relative uncertainty in the main surface aerosol layer (α_aer_ > Δ_DL_) retrieved by the SCC from PRR and VRR signals for cases N1, N2 and D1. Black plus signs (right axis) are the ratio PRR to VRR. For layer-mean values exceeding the selected vertical scale the numerical value is reported in black on the top of the bar.

The statistical uncertainty of a product *X* (α_aer_ or β_aer_) is noted σ_X_. The relative uncertainty is computed as σ_X_/*X*. A low relative uncertainty (~10%) is an indicator of a reasonably good estimation [32]. The only Raman product that depends only on the Raman channel signal is the extinction coefficient. For comparison purposes it is the product chosen in Figure 9 and Figure 10. Figure 9 shows the profiles of α_aer_, EVR, σ_αaer_ and σ_αaer_/α_aer_ retrieved from both PRR and VRR signals. Since σ_αaer_ and σ_αaer_/α_aer_ for PRR and VRR channels were not calculated with the same vertical resolution, they are not directly comparable. To solve this issue, we have estimated the σ_αaer|VRR_ that ELDA would have calculated if the vertical resolution had been that of PRR.

To do so, we use the property that the standard deviation of the estimation of a slope (the method employed by the Raman algorithm) through a linear fit regression is inversely proportional to (N(N^2^ − 1))^1/2^, being N the number of samples used in the estimation and assuming that the noise is statistically the same in all samples [33]. For large values of N, such that N^2^»1, this standard deviation is proportional to N(N)^1/2^ = N^3/2^. With the native range resolution of the EARLINET/UPC lidar system of 3.75 m, typical EVR calculated by the SCC in the range 100–2000 m are obtained by averaging over a number of 26 to 534 samples. This number can be considered large, hence equivalent VRR σ_αaer_ and σ_αaer_/α_aer_ can be calculated at EVR_|PRR_ by multiplying the original profiles by the profile of (EVR_|VRR_/EVR_|PRR_)^3/2^.

The recalculated or normalized profile is labelled “VRR normalized” in the legend of Figure 9. Figure 10 shows histograms with layer-mean values of EVR, σ_αaer_ and σ_αaer_/α_aer_ of both PRR and VRR retrievals in the main surface aerosol layer (α_aer_> Δ_DL_). In case D1 the layer-mean value was calculated between the ground and 2.2 km, which is the maximum height up to which α_|VRR_ is defined. For comparison purposes, the layer-mean values of σ_α_ and σ_α_/α of the VRR retrievals in Figure 10 are averages of the normalized magnitudes. For each magnitude (EVR, σ_αaer_ and σ_αaer_/α_aer_) a reduction factor is obtained dividing the PRR retrieval by the VRR one.

In the following discussion (Figure 9 and Figure 10) the VRR retrievals of σ_αaer_ and σ_αaer_/α_aer_ are the normalized ones, i.e., the ones recalculated at EVR_|PRR_. In all cases and at all height ranges, the absolute uncertainty σ_αaer|PRR_ is smaller than the normalized σ_αaer|VRR_ (Figure 9). In the first few hundred meters near the surface, σ_αaer|PRR_ and the normalized σ_αaer|VRR_ are similar for all cases, but they start rapidly to differ and the difference between them increases with increasing height (especially in case D1). In both nighttime cases, above the first few hundred meters, the normalized σ_αaer|VRR_ is larger than σ_αaer|PRR_ but both profiles have a similar shape and no significant degradation of one compared to the other is observed up to the free troposphere. Contrarily, in case D1, the normalized σ_αaer|VRR_ increases strongly above 1.2 km up to values larger than 100 Mm^−1^, whereas σ_αaer|PRR_ stays below 15 Mm^−1^. This degradation of the statistical error of σ_αaer|VRR_ is related to the degradation of the SNR in the VRR channel mentioned in the former section and affects the quality of the retrieval already at 1.2 km within the main surface aerosol layer.

The relative uncertainty σ_αaer_/α_aer_ is also generally smaller for the PRR retrieval than for the normalized VRR one (Figure 9), although they can also exhibit similar values (e.g., range 2.4–3.2 km in case N1). In cases N1 and N2, below 1.5 km, σ_αaer_/α_aer_ for the PRR (and normalized VRR) retrievals are roughly constant and equal to 7 (14) and 8% (17)%, respectively. Above 1.5 km the relative uncertainty increases for both retrievals.

More variations are observed on the VRR retrieval. In case D1, below 1.5 km, σ_αaer_/α_aer_ for the PRR is relatively constant and stays below the quite reasonable value of 10%. This is a promising result for future daytime PRR retrievals. Above 1.5 km, σ_αaer_/α_aer_ increases to values of 30–40%. The normalized VRR σ_αaer_/α_aer_ increases quickly (from the very bottom of the profile) and very strongly (σ_αaer_/α_aer_ > 60% at 0.9 km) and features abrupt fluctuations, sometimes well above 100%.

We now examine the layer-mean values of EVR, of σ_αaer_ and σ_αaer_/α_aer_ in the main surface aerosol layer (α_aer_ > Δ_DL_). We observe a small degradation of EVR between 3 h (N1) and 1 h (N2) temporal resolution (Figure 10a): EVR_|PRR_ increases 7% and EVR_|VRR_ 4% from N1 to N2. This non-linear degradation of both techniques makes PRR retrievals more effective for long time resolution (EVR_|PRR_/EVR_|VRR_ is 0.83 for N2) than for shorter time resolution (EVR_|PRR_/EVR_|VRR_ is 0.85 for N2). The most important EVR reduction occurs for the daytime case. The absolute (relative) uncertainty for the PRR retrieval is quite acceptable: 3.1 (9.3), 4.5 (11.0) and 6.3 (13.2%) Mm^−1^ for N1, N2 and D1, respectively. The normalized VRR σ_αaer_ is more than double for cases N1 and N2, and much larger for D1 (Figure 10b).

The corresponding σ_αaer_/α_aer_ are 24.2, 19.3 and 94.0% (Figure 10c), respectively. We conclude that the relative uncertainty of the PRR retrieval for 3-h nighttime measurements is roughly three times smaller than the VRR one. For 3-h daytime measurements it is roughly seven times smaller than the VRR one. Again, the highest reduction occurs for the daytime case.

Several attempts to retrieve aerosol extinction and backscatter via PRR implementations have been made in the recent past. As part of the discussion we aim to compare our results with some recent bibliographic endeavors, some of which have already been used for comparison in the temperature analysis Section 2.4. This section is focused on the measurements and the products obtained by [11] at 355 nm, [7] at 532 nm and [21] at 1064 nm.

The recent work of [11] is the most similar to ours, achieving aerosol extinction and backscatter retrievals with a PRR channel at 355nm. Both daytime and nighttime measurements were made with 1-h and 1-min. temporal resolutions, respectively. For nighttime study cases they were able to retrieve aerosol extinction in a 1.4–3.5 km range (main aerosol layer).

1-min time resolution profiles are compared to a 30-min integrated profile, finding variations of less than the 30% between products. For daytime study cases, extinction and backscatter were retrieved between 1.4 and a top height varying between 2.5 and 3.0 km. The only significant uncertainty considered is due to temperature, which has been compared in Section 2.4. The main differences of our work with respect to [11] are temporal resolutions. For our daytime study case, three hours temporal resolution permitted to retrieve aerosol extinction in a wider range, from 0.35 to 4 km. For nighttime study cases we could not reduce the temporal resolution below 1 h. Nevertheless, considering the reported variability between 1-min. and the averaged 30 min. profiles, we can infer that 1-h profiles can give a quite accurate glimpse of the overall aerosol load in a regular measurement.

## 5. Conclusions

A pure rotational Raman channel at 353.9 nm has been implemented in the EARLINET/UPC multi-wavelength lidar system. This new channel detects backscattered signals produced by the PRR effect of atmospheric N_2_ and O_2_ excited by the emission wavelength of 354.7 nm. Spectral filtering was obtained by cascading two extremely narrow and steep interference filters with an equivalent (i.e., for the two filters in cascade) CWL at 353.9 nm, FWHM < 0.8 nm, transmission at peak > 70% and OD8 at 354.7 nm. To study the temperature dependency, the sum of N_2_ and O_2_ differential backscatter cross section weighted by the respective atmospheric concentration and the filter transmittance was calculated as a function of temperature in a range 200–300 K. Variations were smaller than 3% in that range and smaller than 0.5% in the range 230–300 K corresponding to a height range of 0–10 km.

PRR and elastic signals have been inverted for the first time with the EARLINET Single Calculus Chain and profiles of aerosol extinction and backscatter coefficients have been retrieved successfully in daytime and nighttime conditions. To fully quantify the improvement of pure rotational over vibro-rotational Raman signals, simultaneous measurements at the VRR wavelength of 386.7 nm were performed. Two cases were taken with different aerosol loads and vertical structures. The signal-to-noise ratio was found in agreement with the theory: an increase of a factor 2.8 and ~7.6 was observed for 3-h nighttime and daytime measurements, respectively, when using PRR vs. VRR. Improvements in terms of retrieved optical properties are measured in terms of the reduction of the effective vertical resolution, EVR, and of the uncertainties (absolute, σ_αaer_, and relative, σ_αaer_/α_aer_) associated to the extinction coefficient in the main aerosol surface layer when using PRR retrievals vs. VRR ones.

For long (3 h), nighttime measurements EVR is reduced by 17%, σ_αaer_ is divided by 2 and and σ_αaer_/α_aer_ is divided by 3 when using PRR instead of VRR. During daytime and also 3-h measurements EVR is reduced by 20%, σ_αaer_ is divided by 10 and σ_αaer_/α_aer_ by 7 when using PRR instead of VRR. In the daytime case, the VRR extinction coefficient is retrieved in a limited height range (<2.2 km). This prevents the SCC from finding a suitable calibration range in the search height range of 4–8 km for the retrieval of the backscatter coefficient, so the advantage of using PRR instead of VRR is particularly evident in daytime conditions. For short (1 h), nighttime measurements EVR is reduced by 15%, σ_αaer_ is divided by a little more than 2 and σ_αaer_/α_aer_ is divided by a little less than 2 when using PRR instead of VRR.

## Figures and Tables

**Table 1:** Calculated intensities of the Raman backscatter spectra of Pure rotational Raman (PRR) and Vibro-rotational Raman (VRR) for diatomic nitrogen (N_2_) and diatomic oxygen (O_2_).

	Units	N_2_	O_2_
PRR	10^−8^ m^−1^ sr^−1^	12.4423	8.0672
VRR	10^−8^ m^−1^ sr^−1^	0.5866	0.2178

**Table 2:** Description of the optical elements.

Element	Acronym	Manufacturer Model	Description
**Lens**	L1	Edmund Optics T46-266/T08-058	UV GFS UV-AR coating, PCX D = 25.4 mm, BFL = 33.03 mm,
**Dichroic**	D1	CVI LWP-45-RU407/386/355-TU1064/607/532	Side 1: Ru ≥ 99% @407, 386, 355 nm and Tu ≥ 85% @1064, 607, 532 nm
**Lens**	L2	Edmund Optics T46-271/T08-007	UV GFS UV-AR coating, PCX D = 25.4 mm, BFL = 147.82 mm,
**Beamsplitter**	BS	Melles Griot 03BTQ027	UV GSFS beamsplitter D = 50 mm, Tr = 3 mm
**Dichroic**	D2	CVI SWP-45-RU407-TU355-PW-1525-UV	Side 1: Ru ≥ 98% @407 nm, Tu ≥ 60% @355 nm, Side 2: AR @ 355 nm
**Mirror**	M	Melles Griot 02MFG017	Protected aluminum round flat mirror D = 38 mm, T = 10 mm
**PRR Interference filters**	PRR Filters	Alluxa Custom made	CWL: 353.9 nm, FWHM: 0.8 nm
**VRR Interference filter**	IF1	Barr Custom made	CWL: 386.7 nm, FWHM: 3 nm
**Elastic Interference filter**	IF2	Barr Custom made	CWL: 354.7 nm, FWHM: 1 nm
**Lens**	L3	Edmund Optics T46-292/T08-077	UV GSF UV-AR coating, DCX D = 25.4 mm, BFL = 21.34 mm, CT = 10.9 mm
**Eyepiece**	EP	Edmund Optics	F = 18 mm, d = 15 mm

**Table 3:** Calculated Effective Differential Cross-Sections (EDCS) and Optical Path Loss (OPL) of Pure rotational Raman (PRR) and Vibro-rotational Raman (VRR) lines/channels. The optical path loss is estimated for the entire optical path behind the fiber bundle excluding the interference filters.

	Units	Element	PRR	VRR
EDCS	10^−8^ m^−1^ sr^−1^	-	3.5065	0.4252
		-	(N_2_ and O_2_)	(N_2_)
OPL	Fraction	L1	0.9	0.9
		D1	0.99	0.99
		BS	0.5	0.5
		L2	-	0.9
		D2	-	0.98
		L3	0.9	0.9
		EP	0.9	0.9
		OPL	0.36	0.32
(EDCS)(OPL)	10^−8^ m^−1^ sr^−1^	-	1.2623	0.1360

**Table 4:** Temperature dependence comparison with previous works. Temperature range: 230–300 K. Aerosol load of 100 Mm^−1^.

	Veselovskii (2015)	Haarig (2016)	Ortiz (2020)	This Work
	*λ*_0_ = 532 nm	*λ*_0_ = 1604 nm	*λ*_0_ = 355 nm	*λ*_0_ = 355 nm
*ψ*(*T*)	<1 %	<4%	--	<0.5%
*X_β_*	<1 %	--	<4%	<1%
Δα	<2 Mm^−1^	--	<−1.6 Mm^−1^	<1 Mm^−1^
Δα (%)	<2%	--	<1.6%	<1%

**Table 5:** Main characteristics of the measurements used in this work. The AERONET measurements are level 1.5.

Case	Units	N1	N2	D1
**Conditions**		Nighttime	Nighttime	Daytime
**Date**		11/3/2020	11/2/2020	21/3/2020
**Start time**	UT	18:57	18:57	12:46
**Temporal resolution**	Hours	3	1	3
**Nearest AERONET**				
**Time**	UT	17:05	17:05	12:59
**AOD_440_**		0.15	0.15	0.26
**AE_440–675_**		1.27	1.27	0.71
**Probable airmass origin**		Local	Local	Local, dust in the FT

## Data Availability

The data presented in this study are available on request from the corresponding author.

## Appendix A. Pure Rotational and Vibro-Rotational Raman Differential Backscattering Cross-Section of N_2_ and O_2_ Calculation

Selection rules for nitrogen (N_2_) and oxygen (O_2_), are Δν = 0, ±1 for the vibrational quantum number and ΔJ = 0,±2 for the rotational quantum number. The Differential Backscatter Cross Section (DBCS) at an excitation wave number ν_0_ = 1/λ_0_ and temperature T is:

(A1) $$\sigma^{i}{}_{PRR,VRR}\left(J,T\right)={\left(2\pi \right)}^{4}\cdot {\left[\nu_{0}-{\left|\Delta \nu \left(J\right)\right|}^{i}{}_{PRR,VRR}\right]}^{4}\cdot \frac{g_{N}\cdot \Phi^{i}{}_{PRR,VRR}\left(J\right)}{{\left(2I_{N}+1\right)}^{2}\cdot \xi }\cdot \exp\left[\frac{hcB_{0}\cdot J\left(J+1\right)}{kT}\right]\;\begin{array}{cc} i=S,Q\to J=0,1,2,3,\ldots & i=O\to J=2,3,4,\ldots \end{array}$$

Subindex *i* = 1, 2 correspond to the molecules N_2_ or O_2_. *O*, *Q* and *S* stand for the spectrum branches. ξ ≈ *k**T*/2*hc**B*_0_ is the partition function, *h* is Planck’s constant, *k* is Boltzmann constant and *c* is the speed of light.

Molecular constants are shown in Table A1, Table A2 and Table A3. Data compiled from [14,16,34,35,36,37].

**Table A1:** Statistical weigh factor g_N_ and nuclear spins I_N_ values for N_2_ and O_2_.

	g_N_	I_N_
N_2_	6 for J even; 3 for J odd	1
O_2_	0 for J even; 1 for J odd	0

**Table A2:** Ground-state rotational and centrifugal distortion constants.

	B_1_ [m^−1^]	B_0_ [m^−1^]	D_0_ [m^−1^]
N_2_	197.219	198.957	5.76 × 10^−4^
O_2_	142.188	143.768	4.85 × 10^−4^

For the vibro-rotational contribution which is displaced an amount *ν_vib_* from the excitation wavelength number *ν*_0_, the frequency shifts are given by:

(A2) $$\begin{array}{cc} {\left|\Delta \nu \left(J\right)\right|}_{VRR}^{S}\approx \nu_{vib}+\left(4J+6\right)B_{1}, & J=0,1,2,\ldots \end{array}$$

(A3) $$\begin{array}{cc} {\left|\Delta \nu \left(J\right)\right|}_{VRR}^{Q}\approx \nu_{vib}+J\left(J+1\right)\left(B_{1}-B_{0}\right) & J=0,1,2,\ldots \end{array}$$

(A4) $$\begin{array}{cc} {\left|\Delta \nu \left(J\right)\right|}_{VRR}^{O}\approx \nu_{vib}-\left(4J-2\right)B_{0}, & J=2,3,4,\ldots \end{array}$$

For each molecule, their specific vibrational wavenumber is *ν_vib_* = 2330.7 cm^−1^ and *ν_vib_* = 1556.4 cm^−1^ for N_2_ and O_2_, respectively. Phi functions are defined as:

(A5) $$\begin{array}{cc} \Phi_{VRR}^{S}\left(J\right)=\frac{h}{8\pi^{2}c\nu_{vib}\cdot \left[1-\exp\left(-hc\nu_{vib}/kT\right)\right]}\cdot \frac{7\left(J+1\right)\left(J+2\right)}{30\left(2J+3\right)}\gamma {’}^{2}, & J=0,1,2,\ldots \end{array}$$

(A6) $$\begin{array}{cc} \Phi_{VRR}^{Q}\left(J\right)=\frac{h\cdot \left(2J+1\right)}{8\pi^{2}c\nu_{vib}\cdot \left[1-\exp\left(-hc\nu_{vib}/kT\right)\right]}\cdot \left[\alpha {’}^{2}+\frac{7\left(J+1\right)\left(J+2\right)}{45\left(2J-1\right)\left(2J+3\right)}\gamma {’}^{2}\right], & J=0,1,2,\ldots \end{array}$$

(A7) $$\begin{array}{cc} \Phi_{VRR}^{O}\left(J\right)=\frac{h}{8\pi^{2}c\nu_{vib}\cdot \left[1-\exp\left(-hc\nu_{vib}/kT\right)\right]}\cdot \frac{7J\left(J-1\right)}{30\left(2J-1\right)}\gamma {’}^{2}, & J=2,3,4,\ldots \end{array}$$

For the pure rotational contribution, a few considerations must be taken; as there is no excited vibrational state, both Stokes and Anti-Stokes branches spread around the excitation wavelength number *ν*_0_. *Q* branch corresponds to Rayleigh scattering which can be estimate as *σ^Q^_PRR_* (*J*,*T*) = *Q_Rayleigh_* = (2*π*)^4^∙*ν*_0_^4^∙(*a*^2^ + (^7*γ*2^/_180_)) [14]. Also, the coefficient *h*{8*π*^2^*c**ν_vib_*∙[1 − exp(^−*hc**ν*^*^_vib_^*/*_k_**_T_*)]}^−1^ can be approximated to 1 [38]. Therefore, frequency shifts around the excitation wavelength are:

(A8) $$\begin{array}{cc} {\left|\Delta \nu \left(J\right)\right|}_{PRR}^{S}\approx \left(4J+6\right)B_{0}, & J=0,1,2,\ldots \end{array}$$

(A9) $$\begin{array}{cc} {\left|\Delta \nu \left(J\right)\right|}_{PRR}^{O}\approx -\left(4J-2\right)B_{0}, & J=2,3,4,\ldots \end{array}$$

Phi functions are defined as:

(A10) $$\begin{array}{cc} \Phi_{PRR}^{S}\left(J\right)=\frac{7\left(J+1\right)\left(J+2\right)}{30\left(2J+3\right)}\gamma^{2}, & J=0,1,2,\ldots \end{array}$$

(A11) $$\begin{array}{cc} \Phi_{PRR}^{O}\left(J\right)=\frac{7J\left(J+1\right)}{30\left(2J-1\right)}\gamma^{2}, & J=2,3,4,\ldots \end{array}$$

Remaining constants are shown in Table A3.

**Table A3:** Molecular constants.

	a^2^ [m^6^/(4πε_0_)^2^]	γ^2^ [m^6^/(4πε_0_)^2^]	a′^2^ [(4πε_0_)^2^ m^2^/kg]	γ′^2^ [(4πε_0_)^2^ m^2^/kg]
N_2_	3.17 × 10^−60^	0.52 × 10^−60^	2.62 × 10^−14^	4.23 × 10^−14^
O_2_	2.66 × 10^-60^	1.26 × 10^−60^	1.63 × 10^−14^	6.46 × 10^−14^

For LiDAR applications, the Raman Backscatter spectrum of N_2_ and O_2_ is calculated by weighting the DBCS by the atmospheric concentration *N*, of either N_2_ (0.7808) or O_2_ (0.2095) [16] as:

(A12) $$\beta_{R}^{i}=N\cdot \sigma_{PRR,VRR}^{i}\left(J,T\right)$$
